# Contrast and Luminance Gain Control in the Macaque’s Lateral Geniculate Nucleus

**DOI:** 10.1523/ENEURO.0515-22.2023

**Published:** 2023-03-17

**Authors:** R. T. Raghavan, Jenna G. Kelly, J. Michael Hasse, Paul G. Levy, Michael J. Hawken, J. Anthony Movshon

**Affiliations:** Center for Neural Science, New York University, New York, New York 10003

**Keywords:** contrast, gain control, LGN, luminance, temporal frequency

## Abstract

There is substantial variation in the mean and variance of light levels (luminance and contrast) in natural visual scenes. Retinal ganglion cells maintain their sensitivity despite this variation using two adaptive mechanisms, which control how responses depend on luminance and on contrast. However, the nature of each mechanism and their interactions downstream of the retina are unknown. We recorded neurons in the magnocellular and parvocellular layers of the lateral geniculate nucleus (LGN) in anesthetized adult male macaques and characterized how their responses adapt to changes in contrast and luminance. As contrast increases, neurons in the magnocellular layers maintain sensitivity to high temporal frequency stimuli but attenuate sensitivity to low-temporal frequency stimuli. Neurons in the parvocellular layers do not adapt to changes in contrast. As luminance increases, both magnocellular and parvocellular cells increase their sensitivity to high-temporal frequency stimuli. Adaptation to luminance is independent of adaptation to contrast, as previously reported for LGN neurons in the cat. Our results are similar to those previously reported for macaque retinal ganglion cells, suggesting that adaptation to luminance and contrast result from two independent mechanisms that are retinal in origin.

## Significance Statement

Visual neurons have a limited dynamic range, so to maintain sensitivity in the face of image fluctuations, the visual system deploys two gain control mechanisms, one for luminance and one for contrast. The responses of neurons in the macaque lateral geniculate nucleus (LGN) are adjusted by these gain controls, which act independently to set sensitivity. The gain control for luminance acts on all neurons, suggesting an origin very early in the visual process. The gain control for contrast predominantly affects neurons in the magnocellular division of the LGN, suggesting a pathway-specific mechanism. The combined actions of these gain controls regulate the firing of LGN neurons to optimally relay visual information from the retina to the visual cortex.

## Introduction

There is substantial variation in both mean light levels [luminance (*L*)] and their local variation [contrast (C)] in natural scenes (Webster and Mollon, 1997; Frazor and Geisler, 2006). Two mechanisms allow the early visual system to adjust to these changes, gain controls for luminance and contrast (Shapley and Enroth-Cugell, 1984). Each mechanism alters the firing of visual neurons to preserve information about the pattern of light on the retina, given wide variations in contrast and luminance and limited neuronal firing rates.

How each adaptation mechanism influences the sensitivity of a cell can differ based on cell class. For the two major ganglion cell classes of the cat retina, for example, contrast gain control exerts a more considerable impact on the responses of Y cells than X cells (Shapley and Victor, 1978). In addition, Y cells become light adapted and transient at lower luminance levels than X cells (Jakiela and Enroth-Cugell, 1976). Similar distinctions hold for the ganglion cells that project to the magnocellular (M) and parvocellular (P) layers of the primate lateral geniculate nucleus (LGN; which we will term M and P ganglion cells), as well as activity for the cells within these layers (which we will term M and P cells). Contrast gain control has more effect on M ganglion cell responses (Benardete et al., 1992; Benardete and Kaplan, 1999). Moreover, as in cat Y cells, M ganglion cells become light-adapted and transient at lower light levels than P ganglion cells (Purpura et al., 1990).

M and P pathway cells also differ in ways that X and Y pathway cells do not. Response gain, the initial slope of firing rate as a function of contrast, is higher in the M pathways than the P pathways for ganglion cells in the retina and recipient M and P layers in the LGN (Derrington and Lennie, 1984; Kaplan and Shapley, 1986; Movshon et al., 2005). Moreover, while M ganglion cells maintain a significant response gain at scotopic light levels, P ganglion cells have negligible response gain at these light levels (Purpura et al., 1988). Neither of these properties distinguishes X from Y cells (Shapley and Enroth-Cugell, 1984; Shapley, 1992). However, within the X and Y cell classes, there is a clear relationship between the size of the center receptive field (RF) mechanism and the strength of luminance and contrast adaptation. As the center size increases, the effective luminous flux (area × intensity) over the center increases, and cells become light adapted at lower light levels and have higher contrast gain (Enroth-Cugell and Shapley, 1973; Shapley and Enroth-Cugell, 1984). Differences in receptive field size might account for the differences in contrast gain between M and P ganglion cells (Purpura et al., 1988). M ganglion cells have larger dendritic fields than P ganglion cells (Dacey and Petersen, 1992). Measurements of the RF center in both the retina and LGN have shown that M ganglion cells and M layer LGN cells have a larger RF center than retinal and LGN P layer cells (De Monasterio and Gouras, 1975; Derrington and Lennie, 1984).

Changes in filtering properties parallel the changes in response dynamics caused by luminance and contrast gain control. As contrast levels decrease, cat X and Y cells and monkey M ganglion cells become low-pass temporal filters (Shapley and Victor, 1978; Benardete and Kaplan, 1999).

On the other hand, P ganglion cells exhibit little change in their temporal frequency (TF) selectivity with changes in contrast (Benardete et al., 1992), suggesting that contrast gain control does not significantly impact this pathway. As light levels increase, both M and P ganglion cells exhibit a similar elevation in response gain at high temporal frequencies (Purpura et al., 1990). There appears to be little interaction between the luminance adaptation and contrast gain control for cat X and Y LGN cells at photopic light levels, as changes in luminance and contrast exert separable effects on the temporal frequency tuning of these cells (Mante et al., 2005). It remains unclear whether the same holds in the primate M and P system.

Here we seek to address two gaps in our knowledge about how neurons in the M and P layers of the monkey LGN adapt to luminance and contrast. First, we wanted to know whether LGN neurons adapt their response in the same ways reported for retinal ganglion cells that project to the M and P layers (Kaplan and Shapley, 1984; Lee et al., 1990). Some studies show linear transmission between retinogeniculate output and LGN responses (Lee et al., 1983; Seim et al., 2012). However, paired recordings from the retina and LGN retinal in the cat and monkey demonstrate that the magnitude and efficiency of retinogeniculate transmission depend on stimulus contrast (Kaplan et al., 1987; Scholl et al., 2012; Alitto et al., 2019), a nonretinal form of contrast gain control. That Mante et al. (2005) decomposed LGN X and Y responses into separable luminance and contrast factors suggests that retinal and nonretinal contrast gain controls operate independently of mean luminance. Confirming this finding in the primate M and P pathways is the second principal aim of this work.

Understanding the gain control signatures of the M and P pathways at this level of detail is critical to developing models of visual representations in the retina and thalamus. Moreover, given that the M and P pathways remain partially segregated from the input layers of the primary visual cortex to the early extrastriate cortices (Callaway, 2005; Sincich and Horton, 2005), any complete account of cortical visual representations may need to take into account subcortical gain mechanisms within the M and P pathways. Our findings demonstrate that LGN neurons seem to faithfully represent known changes in retinal input under variations in luminance and contrast, that changes in contrast and luminance exert separable influences on the temporal sensitivity of macaque LGN neurons, and that the influence of each adaptation mechanism varies as a function of both cell classes.

## Materials and Methods

We recorded three visually normal male macaques, a 9-year-old *Macaca nemestrina*, an 11-year-old *Macaca mulatta*, and a 3-year-old *Macaca fascicularis*. We used an anesthetized paralyzed preparation, which has been described in detail previously (Movshon et al., 2005). We induced anesthesia with an intramuscular injection of ketamine HCI (10 mg/kg) and maintained anesthesia during the catheterization of saphenous veins and endotracheal intubation with isoflurane. After placement of the animal into a stereotaxic frame, we maintained anesthesia with an infusion of between 6 and 30 μg/kg/h sufentanil citrate, and neuromuscular blockade with an infusion of 0.1 mg/kg/h vecuronium bromide to limit eye movements. We opened a craniotomy and durotomy to allow a 23 gauge guide tube containing a glass-coated tungsten microelectrode (Merrill and Ainsworth, 1972) to be lowered to a position 5 mm above the LGN; we then advanced the electrode out of the guide needle into the nucleus. Before histologic reconstruction, we confirmed entry to the LGN by noting the onset of brisk time-locked visual activity in response to alternating red–green flicker. In most instances, we assigned recordings to layers of LGN based on the sequence of eye dominance across depths relative to the entry into the LGN, aided by functional criteria (described below). We made electrolytic lesions at points of interest along the microelectrode track for most penetrations. After the experiment, we killed the animal with an overdose of barbiturate (pentobarbital, 65 mg/kg) and perfused it with 4% paraformaldehyde in 0.1 m PBS. After blocking the brain around the LGN, we cut parasagittal sections at 50 μm on a freezing microtome. We reconstructed the electrode tracks based on tissue marks and electrolytic lesions visualized in Nissl-stained sections to determine a laminar assignment for each recording site.

### Visual stimulation

We administered atropine sulfate (1%) drops to each eye to dilate the pupils (typical diameter, 6 mm) and paralyze accommodation, and placed gas-permeable +2D contact lenses in each eye to protect the corneas. Throughout the experiment, we periodically removed the contact lenses for cleaning. We estimated refractive error by direct ophthalmoscopy and refined the estimate by finding corrective lenses that optimized the response of visual units. These supplementary spectacle lenses made the retinas conjugate with a screen 114 cm distant.

We generated and controlled stimuli with an Apple Mac Pro computer. We presented stimuli on a CRT monitor (HP1190) running at a resolution of 1280 × 960 pixels (64 pixels per degree) and 120 Hz. Most stimuli were drifting sinusoidal gratings, which varied in luminance, contrast, and drift rate (temporal frequency).

We located the center of the receptive field of each LGN neuron with hand-controlled targets. We then measured the contrast response, spatial and temporal frequency tuning, chromatic selectivity, and size tuning of each cell in separate experiments that varied one parameter at a time. We used the phase of the modulated response of each cell to stimuli with a low spatial frequency (0 or 0.1 cycles/degree) to classify each cell as ON or OFF. In the main experiment, we recorded the response of each neuron to large-field (20° × 15° of visual angle) sinusoidal drifting gratings of a spatial frequency near the optimum of each cell (typically ∼1 cycles/degree). We modulated the temporal frequency of these gratings over time—it either increased exponentially over 14 s from 0.5 to 32 Hz or increased in equal ratio steps every 1.6 s from 0.5 to 40 Hz. We refer to the first stimulus as a “chirp sweep” and the latter as a “stepped sweep.” We presented each sweep at three luminance levels and four or five contrast levels.

Each block began with a blank screen of a given luminance displayed for between 1 and 1.6 s to allow cells to adapt. Five blocks of sweeps were run consecutively at the same mean luminance level, with contrast increasing by 0.5–1.0 octaves (a factor of √2 – 2) over the stimulus presentation interval of 14–24 s. Following the completion of five blocks, the mean luminance increased or decreased in the next block, and we made another set of contrast response measurements. We presented four to five contrast levels for each of the three mean luminances tested, with five repeats per block.

We used contrasts between 0.0125 and 0.8 and luminances between 3.5 and 41 cd/m^2^ (estimated retinal illuminance, 100-1150 trolands (Td)) based on the range of stimulus luminance offered by our CRT display monitor and the precision offered by the bit depth of the display controller. At the lowest luminance, contrasts were between 0.05 and 0.8; at the highest, contrasts were between 0.0125 and 0.4.

### Fitting linear–nonlinear model

We computed averaged response histograms and low-pass filtered them with a second-order Butterworth filter with a 40 or 60 Hz cutoff, depending on the stimulus. For chirp stimuli, the high-pass cutoff was 40 Hz, and for stepped stimuli, 60 Hz, to avoid attenuating neural modulations at the highest temporal frequencies tested (32 and 40 Hz, respectively). We used the entire time course of the response for each combination of contrast and luminance to fit a linear–nonlinear (LN) model and used these models to estimate the response of each cell to temporal frequency as a function of contrast and luminance.

### Model components

The linear component of the model was a cascade of high-pass and low-pass filters introduced by Shapley and Victor (1981). In the frequency domain, this model takes the following form:

(1) $$K\left(s\right)=Ae^{-sD}\left(1-\frac{H_{s}}{1+s\tau_{S}}\right){\left(\frac{1}{1+s\tau_{L}}\right)}^{N_{L}},$$

where A is the amplitude, D is an initial delay, $\tau_{S}$ and $\tau_{L}$ are time constants of the low-pass and high-pass filters, $H_{S}$ is the high-pass strength, $N_{L}$ is the number of stages in the low-pass filter, and s=iω, where ω is the temporal frequency. The first term of this equation is the effect of a temporal delay in the frequency domain, while the second and third terms correspond to a high-pass and low-pass filters, respectively.

We multiplied the linear model by the stimulus S in the frequency domain, S(ω)⋅K(ω), and then inverse Fourier transformed the result to yield the filter output k(t) for a given set of model parameters. We refer to this filter output as the generator signal following Chichilnisky and Kalmar (2002), which approximates the stimulus-driven membrane voltage. The generator signal k(t) passes through a nonlinearity to generate spike rate over time r(t). In this analysis, we used a rectifying nonlinearity, defined as follows:

(2) $$r\left(t\right)=\lfloor k\left(t\right)+k_{0}\rfloor {}^{+},$$

where ${\left\lfloor \right\rfloor }^{+}$ indicates rectification so that:

(3) $$\lfloor x\rfloor^{+}=\{\begin{array}{c} x\text{ if }x>=0\\ 0\text{ if }x<0 \end{array},$$

and $k_{0}$ is a scalar representing the difference between the resting potential $V_{\text{rest}}$ and the threshold membrane potential $V_{\text{thr}}$ (Mechler and Ringach, 2002). Positive values of $k_{0}$ allow the model to have spontaneous firing rates in the absence of visual stimulation. In control analyses, we introduced an exponent on this nonlinearity, but in almost every case tested, the best-fitting exponent was close to 1. Since including an exponent as a parameter did not improve the fit substantially, we used the more straightforward form. The linear and nonlinear components of the LN model were fit separately.

### Fitting procedure for the nonlinearity

We assumed a fixed nonlinearity at each luminance condition, which was estimated as follows. We measured the baseline firing rate at zero contrast for each luminance level. Preceding each run of the sweep stimuli described above, we presented a zero contrast blank screen at one of three luminance levels for 1–1.6 s. We gathered 25–40 s of zero contrast data per luminance level across five repeats and five contrast conditions. We formed a distribution of the binned firing rates at each luminance level. We assumed that the distribution of firing rates observed at zero contrast is the result of rectifying $k_{0}$ when k(t)=0. Prior work has shown that a rectified Gaussian can approximate this distribution of firing rates. The mean of this distribution represents $V_{\text{rest}}-V_{\text{thr}}$, or $k_{0}$ (Carandini, 2004; Mante et al., 2005). We estimated the mean and SD of a rectified Gaussian distribution that minimized the negative log-likelihood of observing the distribution firing rates at zero contrast. This calculation was performed separately at each luminance level (three nonlinearities total), and $k_{0}$ was set equal to the estimated mean.

### Fitting procedure for the linear filter

Across stimulus conditions, the fitting procedure allowed all linear filter parameters to vary. We used the linear filter parameters for M and P cells published in Benardete and Kaplan (1997, 1999) to set bounds on these parameters. We fit the LN model to both the time-varying mean firing rate and the mean of the measured first harmonic (F1) responses in each condition for both stepped and chirp sweep stimuli. The F1 is the amplitude and phase of the response of a cell at the temporal frequency of the stimulus. The F1 of a rectified linear filter (LN) model is derived as follows, according to Mechler and Ringach (2002). Given the difference between the resting membrane potential and the threshold membrane potential $V_{\text{rest}}-V_{\text{thr}}=k_{0}$ and the amplitude response of a linear filter |K(ω)|, the first harmonic response is calculated from the following ratio:

(4) $$\text{χ}\left(\text{ω}\right)=-\frac{k_{0}}{\left|K\left(\text{ω}\right)\right|},$$

such that:

(5) $$|F1(\text{ω})|=\{\begin{array}{cc} \left|K\left(\text{ω}\right)\right| & \text{χ}\left(\text{ω}\right)<-1\\ \frac{\left|K\left(\text{ω}\right)\right|}{\pi }Z\left(\text{χ}\left(\text{ω}\right)\right) & -1\leq \text{χ}\left(\text{ω}\right)\leq 1\\ 0 & \text{χ}\left(\text{ω}\right)>1 \end{array},$$

(6) $$Z\left(\text{χ}\right)=\left[\cos^{-1}\text{χ}-\text{χ}\sqrt{1-{\text{χ}}^{2}}\right].$$

After calculating the F1 at each temporal frequency, we optimized filter parameters to minimize the sum of squared errors between the F1 response (amplitude and phase) of the model calculated using Equation 1 and the measured F1 of the original data. We weighted the sum of squared errors using the square root of the amplitude response at each temporal frequency. Phase differences have a smaller magnitude than amplitude differences, so we reduced amplitude errors to bring phase and amplitude errors into approximately the same range and let them contribute more equitably to the model fit.

For chirp sweeps, we could not calculate the F1 at <4 Hz. Therefore, we optimized filter parameters to minimize the squared error between the time-varying response and the model prediction in time, weighted by the square root of the firing rate at each point in time. The fitting procedure was additionally constrained to match the F1 response that we could calculate >4 Hz. We found this useful to prevent the model from overestimating the response of cells to high temporal frequencies where noise dominates the response for many cells.

### Descriptive model fitting

Below we describe in detail a set of models whose parameters were fit to the F1 response to spatial frequency, temporal frequency, and contrast of a cell. We fit these descriptive tuning models using standard bounded nonlinear optimization (interior-point algorithm). In most instances, we minimized the weighted sum of squared differences between the model predictions and data, where the weights—on the assumption of Poisson variability—were usually the inverse of the square root of the response at each condition.

### Separability analysis

We followed the methods of Mante et al. (2005) and used singular value decomposition (SVD) to examine whether we could decompose contrast-evoked and luminance-evoked changes in temporal frequency tuning into separable factors. We started with the complex output (both amplitude and phase) of the set of linear filters that fit data gathered at various *L* and *C* conditions. We can write the response at each frequency as $F_{L,C}\left(\text{ω}\right)$, which is a complex matrix that is *L* × *C* conditions in size. We used singular value decomposition to partition this matrix into the form $USV^{\text{′}}$, where the columns of *U* and *V* define vectors, each of which is a contrast-defined and luminance-defined cross section. The outer product of these vectors is the best separable approximation to $F_{L,C}$ at a given temporal frequency ( ω). The magnitude of the diagonal values of S indicates the degree to which a particular pair of columns approximates the data in F. The magnitude of the first diagonal value in S provides an estimate of how well the function $F_{L,C}\left(\text{ω}\right)$ can be approximated by the outer product of two vectors. A separability index (SI) can be defined (Depireux et al., 2001) as follows:

(7) $${\text{SI}}_{L,C}=\frac{s_{1}^{2}}{\sum_{i=1}s_{i}^{2}}.$$

This index is between 0 and 1, with values closer to 1 indicating greater separability. We usually analyzed temporal frequency tuning curves derived from model fits. We also used the SVD directly on the measured F1 response as a control.

Two factors limit the efficacy of SVD in our current work. Since stimulus discretization and monitor gamut limit the presentable contrast range, we could not render stimulus contrasts <0.05 or >0.4 at every luminance level presented. SVD cannot operate on a matrix with incomplete or missing entries. For experiments on P cells, this typically means we operate the SVD on contrast conditions between 0.05 and 0.4, while for M cells, we examined contrast conditions between 0.05 and 0.2, for which we could make measurements at every combination of contrast, luminance, and temporal frequency. A second limitation is that occasionally a contrast level presented is at or below the contrast threshold of the cell in question. This threshold effect was often the case with P cells where the SNR was <1 at these contrasts, and a response average could not be determined that was distinguishable from noise. Such noise is not separable, and its inclusion in our SVD calculation might lead to the spurious conclusion that the response of a neuron is nonseparable. To address this problem, we evaluated model fit by computing the normalized correlation coefficient (Schoppe et al., 2016) between the model fit and data with a criterion of 0.30 as a cutoff. This cutoff agreed best with a visual inspection of firing rate traces and the instances where a condition did not appear different from Gaussian noise. We excluded conditions for which the fit of a cell fell below this criterion value.

Additionally, we excluded cells that did not pass this criterion at all luminance conditions and at least two contrasts. Four of 67 P cells did not meet these criteria. Of the remaining cells, we applied SVD and performed SVD on 27 cells with a 3 × 3 $F_{L}\times F_{C}$ matrix (7 M cells, 20 P cells), 34 cells with a 4 × 3 $F_{L}\times F_{C}$ matrix (17 M cells, 17 P cells), and 2 cells with a 5 × 3 $F_{L}\times F_{C}$ matrix (2 M cells).

### Separable model evaluation

To evaluate the quality of the separable model fit to the neural data, we calculated the mean squared error between the separable and nonseparable model predictions from the raw data. The nonseparable model is the predicted response from independently fitting an LN model to each condition. We derive the separable model from these model fits using singular value decomposition, as described above. We evaluated other methods to compare model predictions, including explainable variance and the normalized correlation coefficient (Schoppe et al., 2016). All methods gave very similar results.

### Estimating the influence of trial-by-trial variability

To quantify the effect of response variability, we estimated the coefficient of variation (CV) of the phasor responses for each cell as a function of stimulus contrast (Croner et al., 1993; Extended Data Fig. 5-1, analysis). We fit a descriptive function to mean CV as a function of stimulus contrast for M and P cells separately, using a double exponential of the following form:

$$\text{CV}\left(c\right)=p_{1}\exp\left(p_{2}c\right)+p_{3}\text{exp}\left(p_{4}c\right),$$

where c is contrast, and $p_{1-4}$ are free parameters. We used this average function to estimate the influence of trial-by-trial variability on measures like the saturation index or separability for each real or simulated cell in the analysis presented below in Extended Data Figure 6-1 and Figure 9.

## Results

We recorded neurons across layers of the LGN in three male anesthetized macaque monkeys using single electrodes. We assigned neurons to magnocellular layers (M cells) and parvocellular layers (P cells) based on eye preference and histologic reconstruction, considering microlesions placed along recording tracks and depth. We excluded cells that we encountered at boundaries between layers or within the S layers beneath magnocellular layer 1 (Kaas et al., 1978; Weber et al., 1983) because the depth, eye preference, or chromatic signature (e.g., blue–yellow opponency) was consistent with the functional characteristics of koniocellular cells in the intercalated layers, as reported by others (Hendry and Reid, 2000). Because we only recorded a small number of these cells (*n* = 12), and they are not the focus of this report, we excluded them in subsequent analysis.

### Contrast responses of M and P cells

We recorded 163 M and P cells and performed an initial characterization of each cell using drifting gratings centered on the receptive field of each cell encountered. At the preferred spatial and temporal frequency of each cell, the response gain of M cells was far greater than that of P cells, as expected for cells in the M and P LGN layers (Shapley et al., 1981; Kaplan and Shapley, 1982; Derrington and Lennie, 1984; Levitt et al., 2001; Movshon et al., 2005) as well as M and P ganglion cells (Kaplan and Shapley, 1986; Lee et al., 1990).

**Figure 1.**
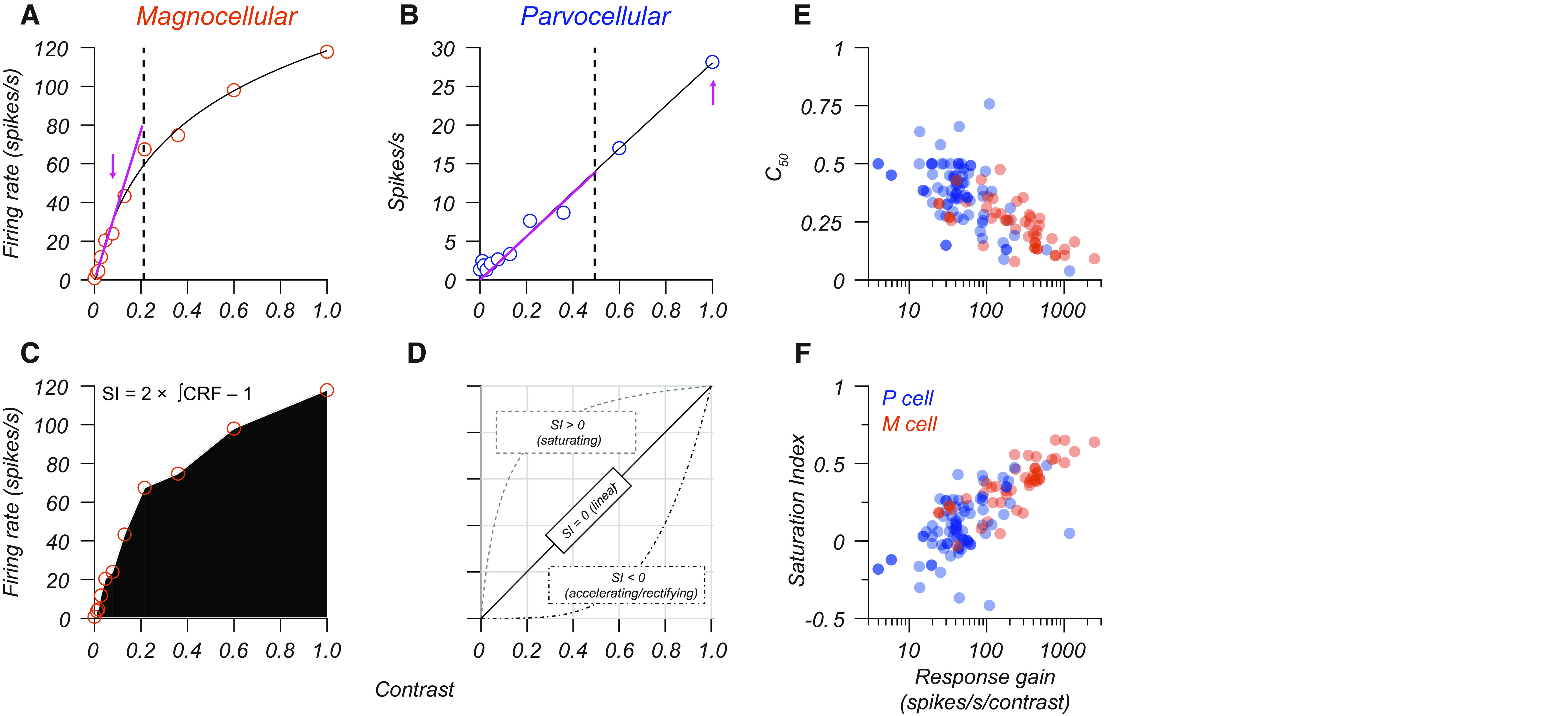
Quantifying response gain signatures of LGN neurons. ***A***, ***B***, Contrast response functions of an example M cell (***A***) and P cell (***B***). Open points are the F1 response at the temporal frequency of the stimulus. Smooth black lines indicate the fit of a descriptive function (Eq. 8) to these data. Dashed lines indicate the C_50_ (M cell, 0.21; P cell, 0.50), and the magenta arrows indicate the maximum contrast within the linear range of each cell at which response gain was calculated (M cell, 387 spikes/s/contrast; P cell, 28 spikes/s/contrast). ***C***, Illustration of the method used to calculate a saturation index. The example M cell has a saturation index of 0.38, while the example P cell has a saturation index of −0.04. ***D***, The relationship between the nature of the contrast response function (accelerating, linear, saturating) and the saturation index. ***E***, ***F***, Response gain versus the C_50_ (***E***) and saturation index (***F***). Blue points represent P cells (*N* = 71), and red points represent M cells (*N* = 41).

We measured the contrast response function of each cell using drifting sinusoidal gratings of near-optimal spatial and temporal frequency, whose contrast varied in logarithmic steps from 0.01 to 1. The open circles in Figure 1, *A* and *B*, illustrate the F1 contrast response function for typical M and P cells. Prior studies suggest three differences in contrast response distinguish M and P cell populations: the contrast level at which a cell reaches half its maximal firing rate (C_50_; Fig. 1*A*,*B*, vertical dashed lines), the slope of the initial rise of the contrast response function (response gain), and the degree to which response saturates as contrast increases. We fit a descriptive model to the F1 response for each cell in our population. The model is a variant of the log contrast response function introduced by Robson (1975). It has the following form:

(8) $$\hat{r}\left(c\right)=r_{\text{offset}}+r_{\text{amp}}\text{log}\left(1+\frac{c}{C_{0}}\right),$$

where $r_{\text{offset}}\leq 0$ allows for a contrast threshold, $r_{\text{amp}}>0$ is a gain term, $C_{0}$ is a saturation constant representing the contrast at which logarithmic saturation begins, and *C* is contrast. Rectification prevents the function from having negative values. Excluding the offset term ( $r_{\text{offset}}$) led to underestimating the response gain and the C_50_ derived from this smooth function, particularly for cells that did not show responses at low contrast levels. We defined response gain as the slope from zero to the maximum contrast ( $C_{\text{sat}}$) at which the response is still linear. $C_{\text{sat}}$ is the maximum contrast tested for linear cells, but it is the saturation constant ( $C_{0}$) for cells that saturate. We computed a saturation index from the raw data as follows:

(9) $$\text{SI}=2\left[\frac{\left(C_{\text{max}}-C_{\text{min}}\right)\int^{}r\left(c\right)}{\left(R_{\text{max}}-R_{\text{min}}\right)}\right]-1,$$

where $R_{\text{max/min}}$ are the maximum and minimum F1 responses, $C_{\text{max/min}}$ are the maximum and minimum contrast presented, and $\int^{}r\left(c\right)$ is the numerical integral of the contrast response function evaluated via the trapezoid method, as illustrated in Figure 1*C*. Figure 1*D* illustrates how this saturation index quantifies contrast response functions that are accelerating, linear, or saturating.

Response gain and SI differed between P and M cell populations, with M cells having higher average contrast gain and more substantial saturation (*t* tests, *p* < 0.0001 for both). The median M cell response gain was five times the response gain of P cells, and the median C_50_ of M cells was 0.6× the C_50_ for P cells. These results broadly match prior reports, except that several P cells in our population had a high response gain (>80 spikes/s/contrast). Further analysis shows that variations in the eccentricity of P cell-receptive fields explain variations in their response gain, a factor we return to in our discussion.

### How contrast and luminance influence responses in the retina

Previous reports have shown that both luminance and contrast gain control maximize sensitivity at high temporal frequencies. Increasing luminance selectively boosts contrast gain at high temporal frequencies in both M and P cells (Lee et al., 1990; Purpura et al., 1990). M ganglion cells exhibit a nonlinear dependence of contrast gain as a function of temporal frequency so that their contrast response function strongly saturates at low temporal frequencies and is linear at high temporal frequencies (Benardete and Kaplan, 1999). P ganglion cells have linear contrast response functions at all temporal frequencies (Benardete and Kaplan, 1997), so increasing contrast by a factor increases the firing rate by the same factor. We, therefore, expect the following for the contrast response functions of M layer and P layer LGN neurons if they follow their retinal inputs, as follows: (1) M cell contrast response functions should saturate at low temporal frequencies and approach linearity at high temporal frequencies; (2) P cell contrast response functions should be linear at all temporal frequencies; and (3) the response gain of M and P cells should increase with luminance, most prominently at high temporal frequencies.

### How contrast and luminance influence responses in the LGN

**Figure 2.**
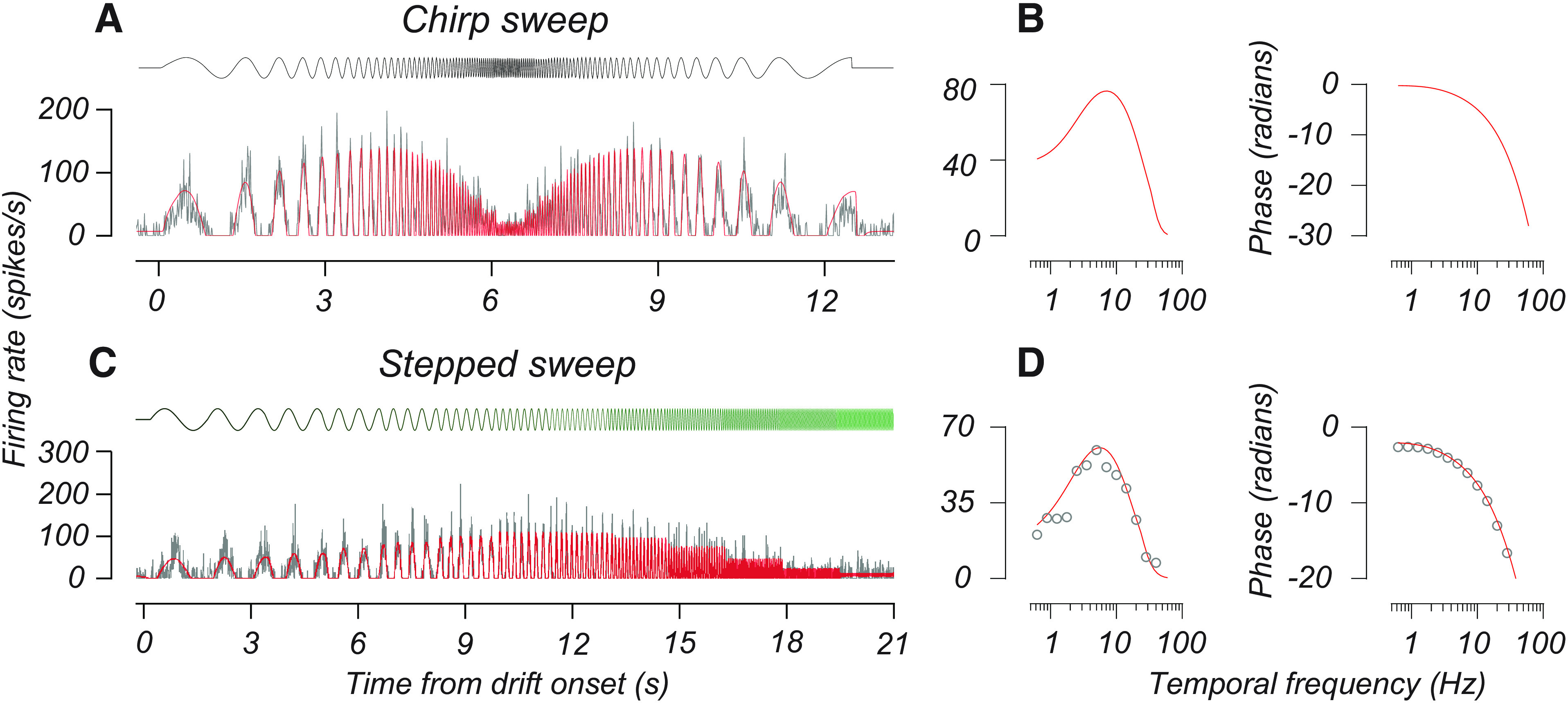
Using sweep stimuli to estimate temporal frequency tuning. ***A***, ***B***, The response of an example M cell to the chirp sweep stimulus. At the top of the figure, the black curve indicates the temporal contrast profile of the stimulus. In ***A***, gray curves plot the firing rate of the cell over time, aligned to drift onset, and red curves are the response predicted by the LN model. The two plots in ***B*** give the amplitude (left) and phase (right) response of this LN model as a function of temporal frequency. ***C***, ***D***, The response of a different M cell to the stepped sweep stimulus. Plots follow the same conventions as in ***A*** and ***B***. The topmost sinusoidal curve in ***B*** indicates the temporal contrast profile of the stepped sweep stimulus, and each epoch is shown on a scale from black to green as the temporal frequency increases. Colors indicate periods of constant frequency (each 1.6 s). The open red points in ***D*** are the amplitude and phase of the first harmonic response calculated from these spike times.

To evaluate whether our population of M and P cells was consistent with prior reports of retinogeniculate inputs, we recorded the response of M and P cells to drifting gratings that varied in contrast, luminance, and temporal frequency. We studied the responses of 67 cells (41 P cells, 26 M cells) with the sweep stimuli (see Materials and Methods), in which temporal frequency varied over stimulus presentation time, as illustrated by the topmost curves in Figure 2, *A* and *C*. Below each stimulus profile, we plot the firing rates of two example cells following the onset of these stimuli. We fit an LN model to these data and used it to estimate the amplitude and phase response of each cell, as illustrated in Figure 2, *B* and *D*.

**Figure 3.**
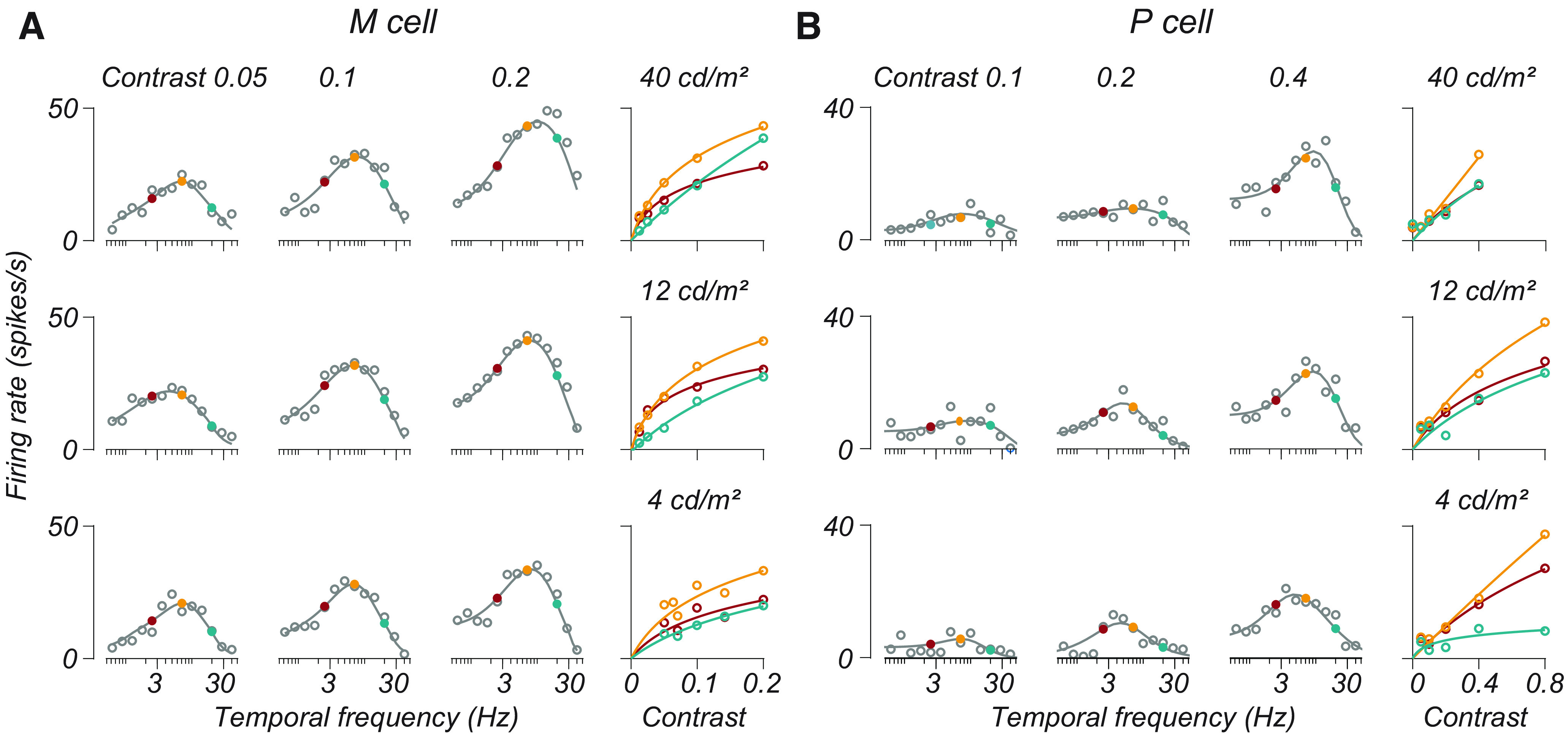
M and P cell temporal frequency tuning as a function of both luminance and contrast. ***A***, ***B***, The responses of one M cell and one P cell at multiple luminances and contrasts. In the main 3 × 3 plot, each subplot is a single set of measurements of temporal frequency tuning at a given contrast and luminance. Contrast values increase left to right, and luminance values increase from bottom to top. Open gray points are measured F1 responses for cells (recorded with the stepped sweep stimulus). Smooth lines through these points plot the amplitude response of the LN model fit to each condition. Filled points indicate temporal frequencies 0 ± 1.5 octaves from the preferred frequency. Colors indicate low (red), medium (gold), and high (teal) temporal frequencies. ***A***, ***B***, The rightmost subplots show the contrast response functions estimated from the LN model at these three temporal frequencies (same color convention) for each luminance. Open points in these subplots are the response of the LN model as a function of contrast. The smooth curves in these subplots show the fit of a descriptive function (Eq. 8) to these data. Temporal frequency tuning curves in the main 3 × 3 plot show only a subset of stimulus contrasts, while the contrast response functions in the subplots indicate the response across all tested contrasts.

Figure 3 illustrates the temporal frequency tuning of two cells (one M and one P) at three luminances (from top to bottom) and three contrasts (from left to right). Open points illustrate the F1 response measured from these cells, and the smooth lines indicate the estimated F1 based on Equation 1. We averaged temporal frequency tuning curves across all conditions for each cell, selected the peak of this curve, and defined it as the “medium” temporal frequency. Frequencies 1.5 octaves below and above the medium temporal frequency were defined as “low” and “high,” respectively.

The filled red, gold, and teal points in Figure 3, *A* and *B*, indicate that these temporal frequencies, for which we plot the contrast response functions. These two cells behaved consistently with the observations of Benardete and Kaplan (1999). The M cell in Figure 3*A* exhibited a saturating contrast response function for low and medium temporal frequencies but was more nearly linear at high temporal frequencies (Fig. 3*A*, rightmost subplots, compare red and gold curves, teal curves). The P cell in Figure 3*B* had a more linear contrast response function at all temporal frequencies. Both cells showed an elevation in response gain for high temporal frequencies as a function of luminance, consistent with the findings of the study by Purpura et al. (1990).

**Figure 4.**
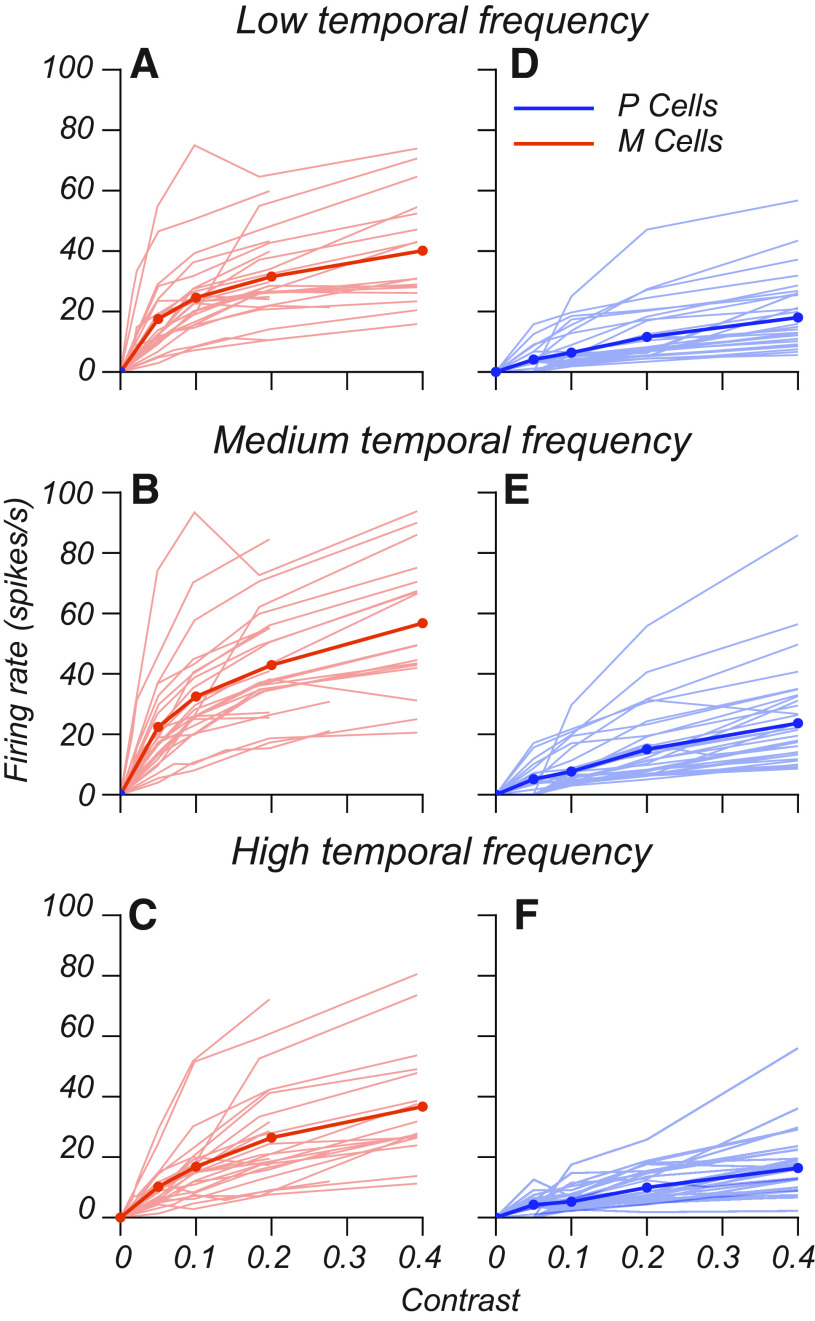
Diversity of contrast response functions within M and P cell populations. ***A–F***, Contrast response functions from M cells (*N* = 26) and P cells (*N* = 37) recorded in the main luminance × contrast experiment. The thin lines in each subplot (***A–F***) represent data for one cell evaluated at contrasts between 0 and 0.4 at one of three temporal frequencies indicated by the title at the top of each subplot. Blue lines represent P cells, and red lines represent M cells. Thick blue and red lines indicate the population-averaged contrast response function. These data are taken from the midluminance condition (10–12 cd/m^2^, 282–356 Td). Given an initial characterization of each cell, the tested contrast ranges in the main experiment differed from cell to cell. Therefore, some M cell curves end at a contrast of 0.2.

Across our recorded population, contrast response functions were broadly similar to these examples, but there was diversity across conditions. Figure 4 plots contrast response functions for all recorded M and P cells at low, medium, and high temporal frequencies at the middle luminance (10–12 cd/m^2^). Both M and P layer cells exhibited a wide range of response gains and degrees of saturation. The thick blue and red curves in Figure 4 are the means. The population-averaged contrast response functions exhibit two clear trends. First, M cells had a higher average response gain and degree of saturation than P cells. Second, the difference between M and P cells was frequency dependent: at high temporal frequencies, the contrast response functions of M cells were more linear (Fig. 4, compare *D*, *E*, initial slope and degree of saturation of the curves, *F*, curve).

Separating population-averaged data by luminance condition revealed that the increased linearity exhibited by M cells at high temporal frequencies is luminance dependent.

**Figure 5.**
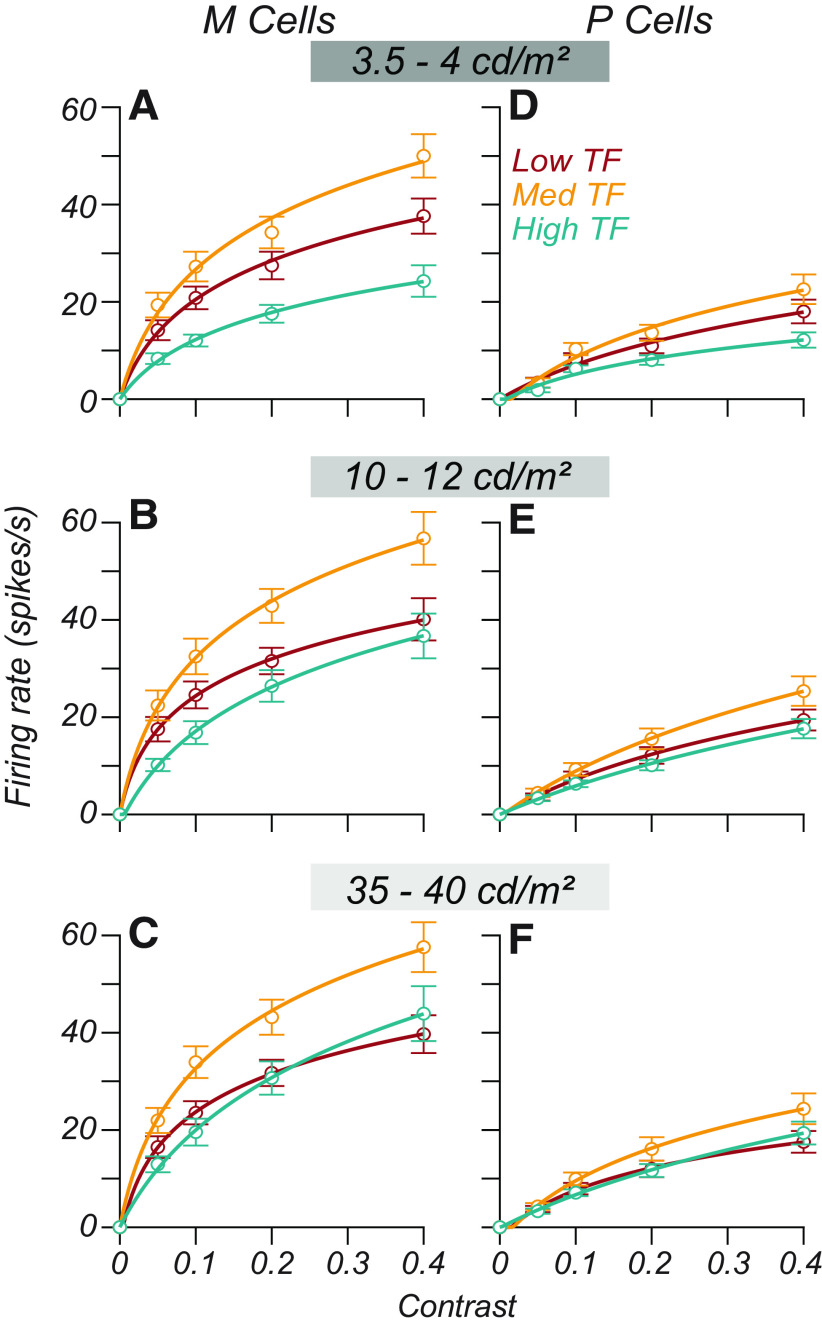
Average contrast response functions as a function of luminance. ***A–F***, The average contrast response function for P cells (***A–C***) and M cells (***D–F***) over a contrast range of 0*–*0.4. The background luminance varies across rows. Low, mid, and high luminance correspond to 3.5, 12.6, and 41 cd/m^2^ for most cells (100, 356, 1159 Td). The smooth curves through the points give the fit of Equation 8 to the data. Error bars are the SEM across cells. The color of each curve indicates the TF. Gold, Medium TF; red, medium TF – 1.5 octaves; teal, medium TF + 1.5 octaves. Average saturation indices across these conditions are shown in Extended Data Figure 5-1.

The thick lines in Figure 5*A–F* indicate the population-averaged contrast response functions of M and P cells at different background luminances. Individual curves in each subplot reflect the contrast response function evaluated at a particular temporal frequency (red, low; gold, medium; teal, high; Fig. 3). For both M and P cells, the contrast response function at high temporal frequencies (Fig. 5*A*,*D*, teal curves) peaks at a low firing rate when stimulus contrast is 0.4, but as luminance increases (from left to right), the teal curves begin to lift upward.

10.1523/ENEURO.0515-22.2023.f5-1 Figure 5-1 Average saturation index across temporal frequencies and luminances. ***A***, ***B***, Saturation indices for M and P cells as a function of temporal frequency. Each error bar is the mean ± SEM of the saturation indices of M cells (*n* = 26) and P cells (*n* = 41) as a function of temporal frequency. The color of each line indicates the luminance level. Lines connect conditions that are statistically different from one another (Wilcoxon rank-sum test, *p* < 0.005). Download Figure 5-1, EPS file.

For M cells, we found that the saturation index was lower (i.e., responses were more nearly linear) at high temporal frequencies. As luminance increased, the average saturation index of M cells grew significantly at low and medium temporal frequencies (Extended Data Fig. 5-1*A*). P cell contrast response functions had similar degrees of saturation across temporal frequency and luminance (Extended Data Fig. 5-1*B*,*D*). As a function of luminance, the average saturation indices of M and P cells were most similar at 4 cd/m^2^ (Extended Data Fig. 5-1, compare *C*, *D*).

**Figure 6.**
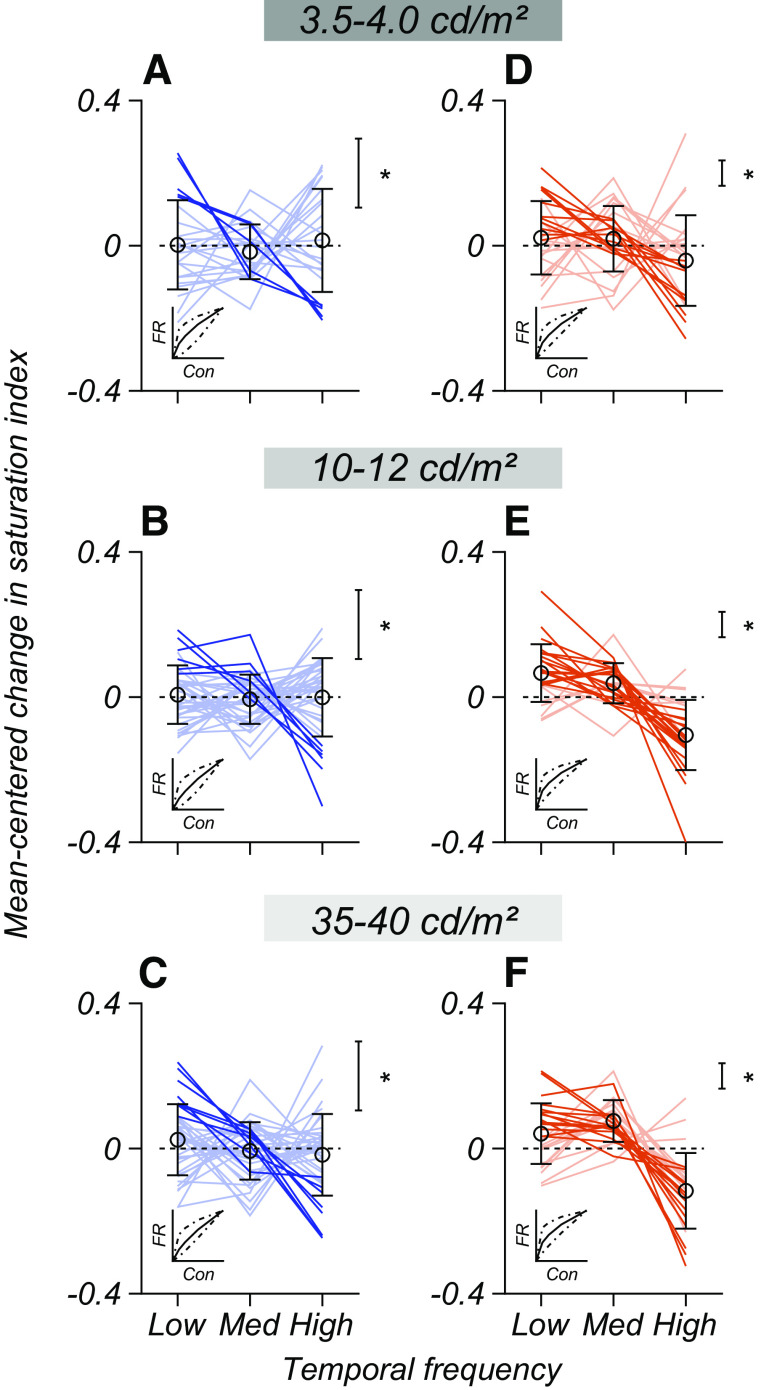
Change in response saturation as a function of luminance and temporal frequency. ***A–F***, Each line shows the change in saturation index across temporal frequency for P cells (*N* = 41), and M cells (*N* = 26). Changes are relative to the average saturation index across frequencies. Open points are the mean (±SEM) saturation index at each TF. The insets in each subplot illustrate the shape of the contrast response function at minimum (dot-dashed), median, and maximum (dot-dashed) saturation indices in each plot. The scale bars marked with asterisks show the difference in saturation index expected by chance for each cell type. Dark lines cross this criterion and show a significant monotonic decrease in saturation as a function of temporal frequency (contrast gain control). Extended Data Figure 6-1 illustrates how chance changes in saturation index were calculated.

We found that these trends were not because of outliers but were characteristic of single cells. The plots in Figure 6*A–F* show the single-cell saturation indices of contrast response functions from low to high temporal frequencies. To estimate the expected effect size and significance, we used a simulation based on the average CV of M and P cell responses to estimate the largest change in saturation index expected by chance for each cell type. Depending on the background luminance, 10–20% of P cells (4–8 of 41) showed a monotonic decrease in saturation index expected from a contrast gain control mechanism (Fig. 6*A–C*, thick lines). By contrast, 42–73% of M cells (11–19 of 26) showed significant contrast gain control, depending on background luminance (Fig. 6*D–F*, thick lines).

10.1523/ENEURO.0515-22.2023.f6-1 Figure 6-1 Simulating the expected change in saturation index on a cell-by-cell basis. ***A***, The variability of the F1 response. The open circles are trial-by-trial F1 responses plotted in the real plane versus the complex plane. The closed circle is the mean F1. The length of the dashed line is the amplitude response of this cell, and the angle of the dashed line from the origin indicates the phase. The SD across trials is measured from the euclidean distances between the single trials and their average. ***B***, ***C***, Coefficient of variation as a function of contrast. Each line is the relationship between the CV (SD/mean F1), and the bolder lines are the average CV as a function of contrast per cell class. The dashed line is the fit of a double exponential to these data. ***D***, Simulations of different contrast response functions. Each line is a single contrast response function that passes through the points (sampled at the contrasts used in our experiment). The darkness of the line indicates how saturated the cell is. ***E***, ***F***, Chance-level variations in the saturation index for P and M cells. Each set of points is a single trial, simulated from the mean responses shown in ***D*** and the empirically derived relationship between contrast and coefficient of variation. Lines through these points show the median and 95% confidence intervals on these data. For each cell class, the significance criterion we selected was the maximum chance-level change in saturation across all degrees of saturation, according to this simulation. Download Figure 6-1, EPS file.

The average response of the contrast response functions of both M and P cells reaches a higher firing rate when both luminance and temporal frequency are high (Fig. 5*C*,*F*). This trend seems consistent with the expectations based on retinal data. Estimates of the slope of the contrast response function proved highly variable because of the confluence of limited contrast conditions and measurement noise, which made it difficult to constrain model parameters during optimization without overfitting or to make substantial assumptions. We, therefore, analyzed the influence of luminance on response gain by considering the firing rate at 0.2 contrast for each cell. This contrast is within the linear range of nearly all the M and P cells we recorded, so if luminance causes an increase in response gain, the firing rate at this contrast should increase as luminance increases.

**Figure 7.**
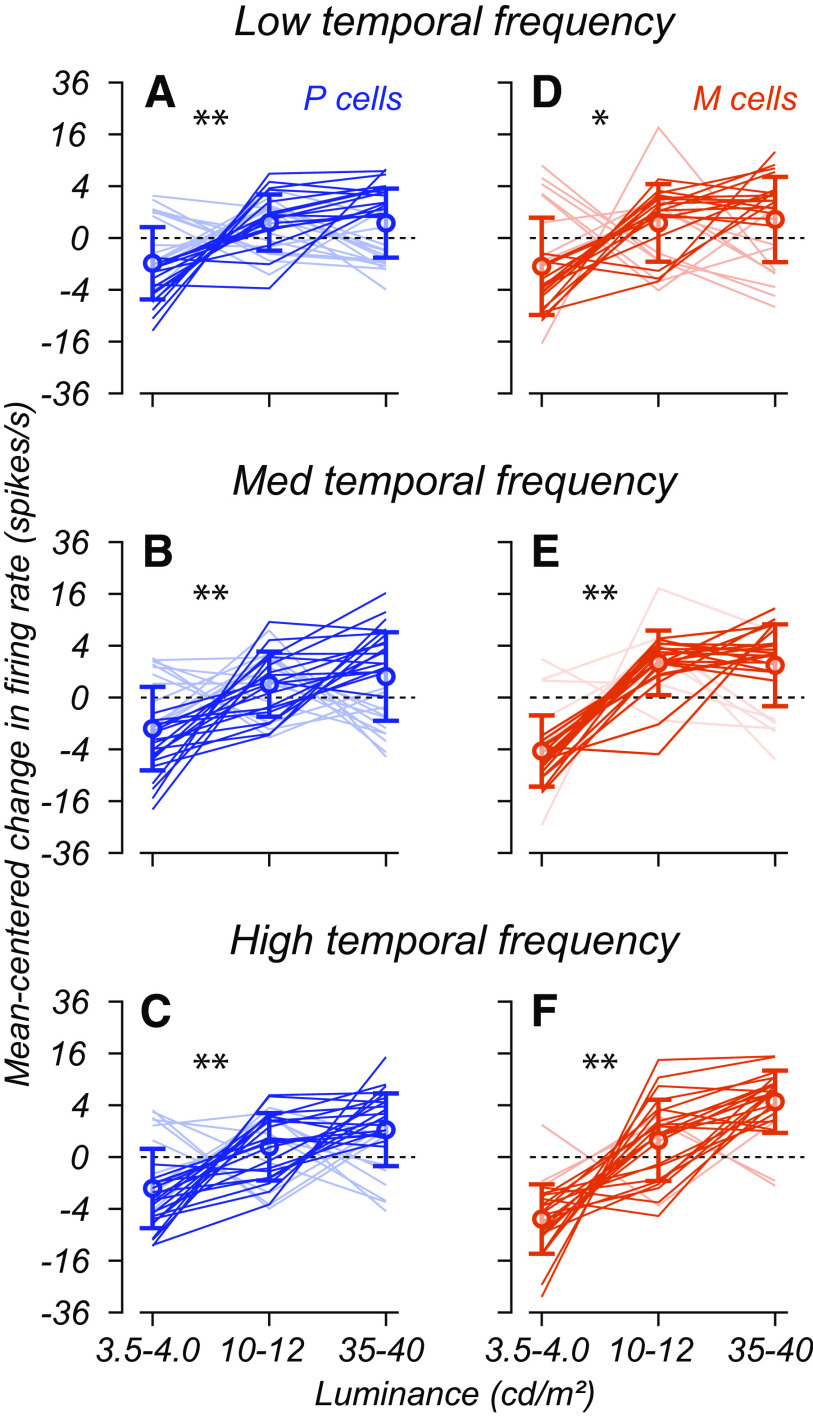
Change in firing rate as a function of luminance and temporal frequency. ***A–F***, Firing rate at 0.2 contrast as a function of mean luminance and temporal frequency. Each line shows the firing rate of a P cell (blue) or M cell (red) at a contrast of 0.2 as a function of background luminance. Each row corresponds to the variation in firing rate at a different temporal frequency, increasing from left to right. We plot firing rates on a square root axis, showing each cell relative to the mean firing rate across luminance conditions. Asterisks indicate significant differences between conditions (*p < 0.005, **p < 0.001, Wilcoxon rank-sum test). Darker lines indicate cells that show a near-monotonic increase in firing rate. Extended Figure 7-1 illustrates the contrast response functions at (3–4.5 vs 35–40 cd/m^2^) and the absolute firing rate differences at multiple contrasts.

The plots in Figure 7*A–F* illustrate the change in firing rate at 0.2 contrast for individual cells as a function of mean luminance. There was a consistent increase in firing rate at 0.2 contrast for both M and P cells as luminance increased from the low (3.5–4 cd/m^2^) to middle (10–12 cd/m^2^) levels we tested (Wilcoxon rank-sum test, *p* < 0.005). This increase in firing rate occurred regardless of the temporal frequency. Between the low and high luminance ranges, average P cell firing rates increased by 5.2 spikes/s at high temporal frequencies and 3.4 spikes/s at low temporal frequencies. For M cells, this effect was greater (low temporal frequency, 4.3 spikes/s; high temporal frequency, 13.1 spikes/s).

10.1523/ENEURO.0515-22.2023.f7-1 Figure 7-1 Contrast response functions at low and high luminance levels for M and P cells. ***A***, ***B***, ***D***, ***E***, Individual cell contrast response functions. Each point is the output of the LN model at that temporal frequency and contrast for either P cells (shades of blue, *n* = 41) or M cells (shades of red, *n* = 26). The smooth lines through the points are the fit of Equation 8 to the data. ***C***, ***F***, Firing rate saturation for individual cells. Each point is the firing rate of a P cell (***C***) or an M cell (***F***) at half-maximum contrast (*x*) versus the firing rate at max contrast (*y*) in response to high-temporal frequency stimuli. Points are shaded according to luminance (darker shades = 3.5–4.0 cd/m^2^; lighter shades = 38–40 cd/m^2^). The marginal distributions along the top show differences as a function of luminance at each contrast condition. The histograms in the corners show differences in firing rate for each luminance condition. Asterisks indicate when the distributions are significant between conditions (Wilcoxon rank-sum test, *p* < 0.005). Download Figure 7-1, EPS file.

In summary, as background luminance increased, the firing rates of both cell types increased across temporal frequencies, with the largest boost occurring at high temporal frequencies. Extended Data Figure 7-1*A–F* illustrates the change in contrast response functions at high temporal frequencies between these luminance conditions. For P cells, the degree of saturation of contrast response functions remained unchanged as luminance increased, but the overall contrast response functions seem to shift upward (Extended Data Fig. 7-1*A–C*). M cells show the same shift upward as a function of luminance (Extended Data Fig. 7-1*D–F*) but also become less saturated, a single-cell confirmation of the trend in average saturation indices illustrated in Extended Data Figure 5-1.

Together these results suggest that cells in the LGN behave similarly to retinal ganglion cells. Only a few P cells show a change in saturation as a function of temporal frequency. They show a small but consistent increase in firing rate at 20% contrast as a function of luminance. M cells show a more substantial increase in firing rate at 20% contrast as luminance increases and become more nearly linear at high temporal frequencies. The firing rate analysis in Figure 7 is consistent with the results of the study by Purpura et al. (1990), who reported that responsiveness increased with luminance.

### Separable influences of contrast and luminance on temporal frequency tuning

These results show that the response gain and saturation of M and P cells change with temporal frequency and mean luminance. The similarity of these results with prior reports of the retinal input to the LGN (Lee et al., 1990; Benardete et al., 1992) suggests that these changes are retinal in origin.

If the adaptation observed in LGN neurons were a consequence of adaptation in retinal inputs, it would predict that mean luminance and contrast changes have independent effects on the amplitude of response at a given temporal frequency for LGN neurons. Mante et al. (2005) tested this explicitly and concluded that in cat LGN, the effects of luminance and contrast on the responses of LGN cells are independent. We extended this analysis to M and P populations in the monkey and decomposed temporal frequency tuning into two separable factors that each vary with luminance or contrast alone.

**Figure 8.**
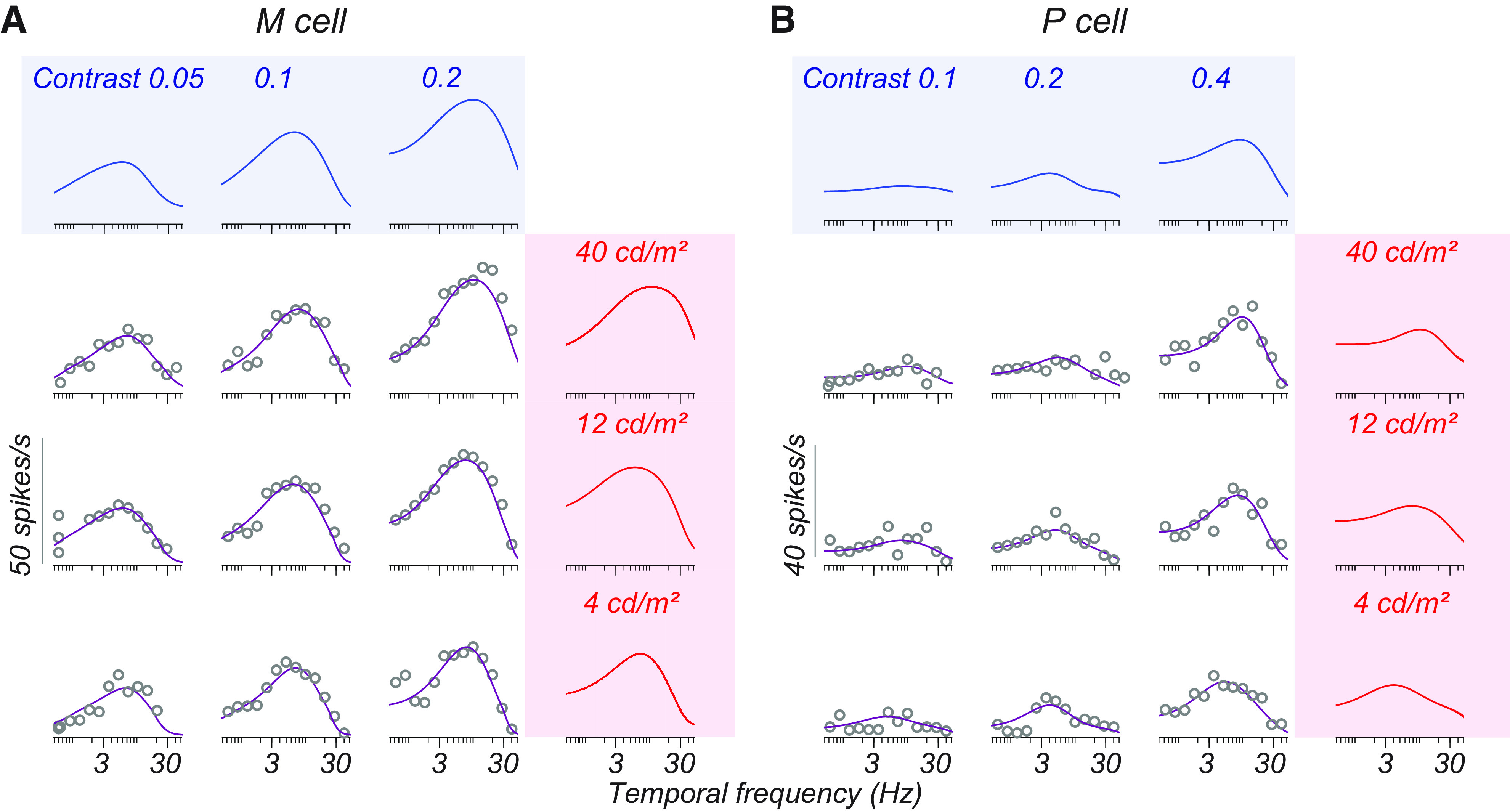
Decomposition of temporal frequency tuning into separable effects of luminance and contrast. ***A***, ***B***, Temporal frequency tuning of example M and P cells for the stepped sweep stimulus and the separable LN model prediction. Gray open points in the area bounded by the shaded boxes are the F1 responses at different temporal frequencies recorded from each cell at a given luminance (row) and contrast (column) condition. These data are those appearing in Figure 3. The solid purple line is the prediction of the separable LN model of the F1 response in each condition. The solid blue and red tuning curves in the shaded boxes are derived using singular value decomposition and represent the independent contribution of contrast and luminance to temporal frequency tuning. The outer product of these tuning curves (purple lines through the data) are the separable function of contrast and luminance that best fit the data.

Figure 8, *A* and *B*, shows the tuning of the same M and P cells initially described in Figure 3. The smooth purple lines running through the data (Fig. 8*A*,*B*, open gray circles) are the product of two sets of tuning curves, one varying by luminance alone and the other by contrast alone. These separable tuning curves (Fig. 8*A*,*B*) appear under shaded blue and red boxes above the points. We estimated these separable functions using SVD, as did Mante et al. (2005). Our analysis begins with a matrix, $F_{L,C}\left(\text{ω}\right)$, that describes the first harmonic response (in the frequency domain) observed at a given value of ω, *C*, and *L*. Each matrix is of size i×j in a given experiment where we test *i* contrasts and *j* luminances. For example, consider the matrix containing the tuning curve data presented in Figure 3. By applying SVD to this matrix, we factorize it into two 3 × 1 separable vectors, $F_{c}\left(\text{ω}\right)$ and $F_{l}\left(\text{ω}\right),$ the product of which yields the best separable approximation to the first harmonic response at that ω value. Considering all temporal frequencies, therefore, we construct two matrices, $F_{C}\left(\text{ω}\right)$ and $F_{L}\left(\text{ω}\right)$, that are ω × *i*, and ω × *j* in size, respectively. Each column of these matrices is a temporal frequency tuning curve that varies as a function of either contrast or luminance. The outer product of columns $F_{C_{i}}\times F_{L_{j}}$ is the separable model approximation to the data recorded in conditions $C_{i},L_{j}$. We refer to this decomposition as the separable LN model. For the two cells shown in Figure 8, *A* and *B*, the separable LN model generated using SVD accounted for 94% of the variance in the data.

**Figure 9.**
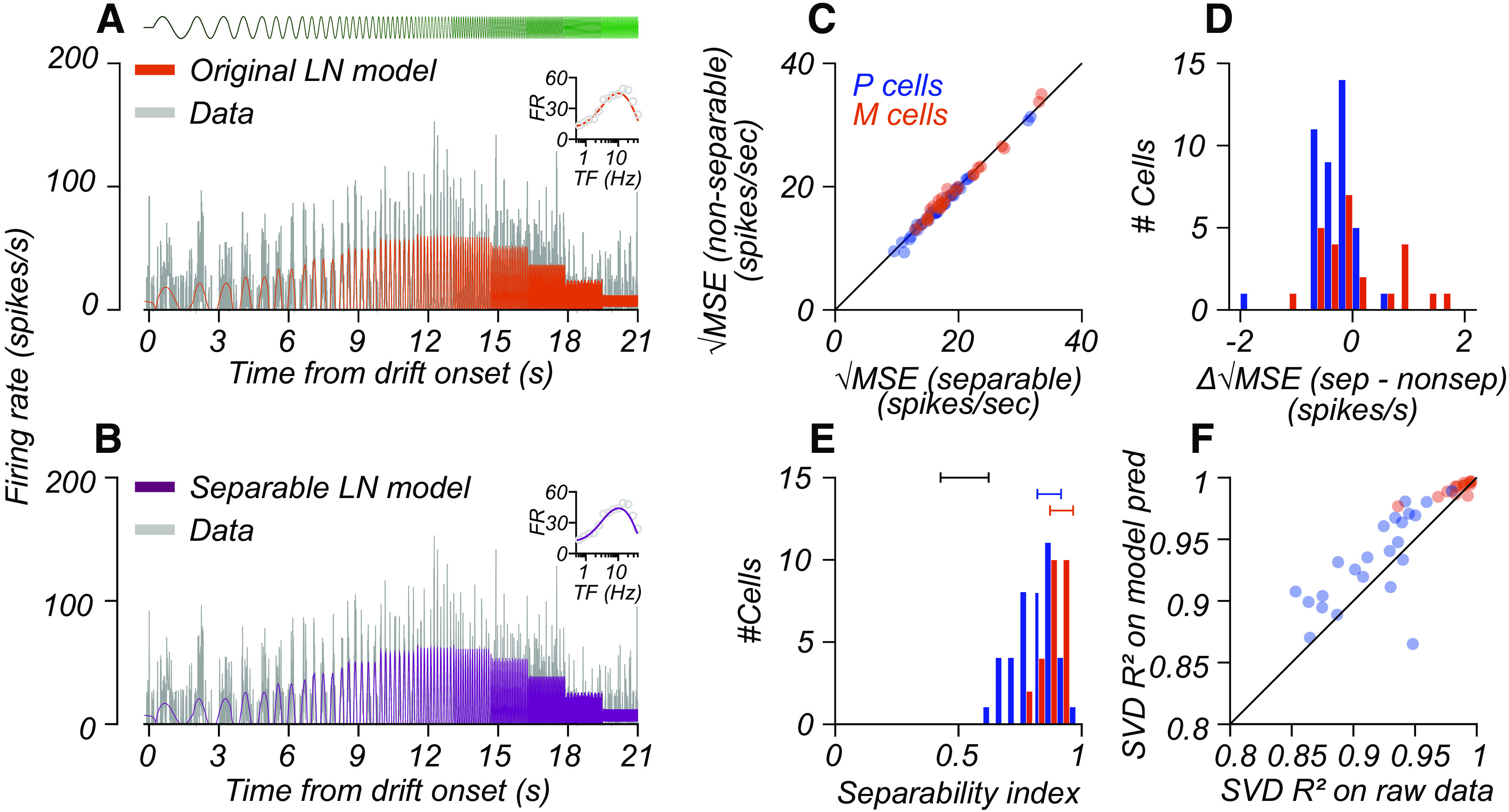
Comparing separable model predictions versus original LN model and F1 responses. ***A***, ***B***, Original and separable LN model predictions for an M cell recorded during a stepped sweep in one stimulus condition. ***A***, ***B***, The gray curves indicate the firing rate of the cell over time and are the same in both panels. The curve at the top of ***A*** indicates the change in stimulus contrast over time. As temporal frequency increases, the curve becomes greener. The solid orange and purple curves inset in ***A*** and ***B*** are the predictions of the original and separable LN models, respectively. The rmse between the original and separable models and the measured response were 15.4 and 15.5 spikes/s, respectively. ***C***, The root mean square error between the original (*x*-axis) or separable (*y*-axis) LN models and the firing rate of the cell. Each point indicates a single cell, and colors indicate cell type, as in previous plots. The black line is unity. ***D***, The distribution of rmse differences between data and the separable model, split by cell type. ***E***, Separability index (see Materials and Methods) split by cell type. The bars at the top of the plot are the estimated ranges of the separability index expected because of uncorrelated data variation (black) and for a perfectly separable neuron with P cell noise (blue) or M cell noise (red). ***F***, Variance explained (*R*^2^) by SVD on raw F1 response versus variance explained (*R*^2^) on F1 responses predicted by the LN model. The data follow the same conventions used in ***C***.

As illustrated in Figure 2, the F1 response in the frequency domain is directly related to the response of a cell in the time domain. By performing SVD in the frequency domain and inverting the separable predictions into the time domain, we generated a prediction of the average firing rate over time in any condition (Fig. 9). In Figure 9, *A* and *B*, the gray curves illustrate the average firing rate over the stimulus presentation period of one condition (41 cd/m^2^, 0.2 contrast) for the M cell of Figures 3*A* and 8*A*. The orange curve in Figure 9*A* shows the fit of the original LN model to these data (before the application of SVD). In Figure 9*B*, the purple curve shows the firing rate prediction generated using the separable LN model. Comparing data in the time domain allowed us to collapse across the datasets where we could compute the F1 directly from the data (the stepped sweep data) and the datasets where we could not (the chirp sweep data). The original and separable models do an equivalent job capturing response for this cell—the root mean square error (rmse) between the data and the prediction of each model is very similar (original model, 15.4 spikes/s; separable model, 15.5 spikes/s). Across the population of recorded cells, the performance of the separable model was essentially indistinguishable from the performance of the original LN model. Consequently, nearly all points lie along the identity line when we plot the rmse for the original LN model versus the rmse for the separable model (Fig. 9*C*). On average, the difference between the separable and nonseparable models was negligible (P cells, −0.27 spike/s; M cells, −0.12 spike/s).

### Controls for model fit quality

We quantify separability in another way in Figure 9*E* by computing a separability index (see Materials and Methods) that is related to the proportion of variance accounted for by the separable model. For our model fits, there was a range of separability indices for each cell type, with a median of 0.81 for P cells and 0.89 for M cells. A limitation of this metric is that limited sampling of data and noise in the response of a neuron causes a reduction in the separability index. At one extreme is simulated data drawn from uncorrelated noise distributions, which are inseparable (Fig. 9*E*, black bar above histograms) and have a low range of possible values. Estimating an upper bound varies from cell to cell, depending on its response and noise levels. For example, we simulated a perfectly separable cell with a response gain of 100 spikes/s/contrast and used the average CV of P cells and M cells (Extended Data Fig. 6-1) to estimate the influence of measurement variability on the separability index. The blue and red bars above the histogram in Figure 9*E* show the bounds of separability indices under these assumptions. However, for cells with lower response gain relative to CV (lower signal-to-noise), the increasing influence of trial-by-trial variability reduces the measurable separability of a cell.

A second limitation of these analyses is that they rely on the fit of a model to generate the separable tuning curves. To see whether this biased the results presented in Figure 9*C*, we analyzed the responses of cells on which we ran a stepped sweep, gathered in two-thirds of our animals (*n* = 42 cells). As noted above, the first harmonic response for this stimulus can be computed directly from spike times without relying on a model. The F1 response at 13 tested temporal frequencies at multiple luminance and contrast conditions yields a matrix, $F1_{L,C}\left(\text{ω}\right)$, similar to the one described above. SVD on this matrix yields two separable matrices, $F1_{C}\left(\text{ω}\right)$ and $F_{L}\left(\text{ω}\right)$, that capture tuning curve shifts driven by contrast or luminance alone and whose product provides the best separable approximation to the raw F1 data. We computed the variance accounted for (*R*^2^) by singular value decomposition applied to either raw F1 data or LN model predictions for those same temporal frequencies. Figure 9*D* illustrates that the SVD accounts for an equal share of the response variance for either set of measurements. This analysis suggests that our LN model-fitting procedure did not bias the SVD toward separable response functions.

## Discussion

Our results show that responses in the magnocellular and parvocellular layers of the nonhuman primate broadly follow the documented properties of retinal inputs to these layers. Luminance and contrast gain control make separable contributions to steady-state responses driven by sinusoidal stimuli. Increasing luminance leads to an elevation and linearization of response gain for high temporal frequency stimuli. The effect of the contrast gain control seems relatively independent of luminance, as saturation for stimuli at or below the peak temporal frequency preference of individual cells occurs regardless of the background light level. Our findings are consistent with the results of retinal recordings (Purpura et al., 1990; Benardete et al., 1992; Benardete and Kaplan, 1999, 1997). The separability of these two processes can be demonstrated directly on temporal frequency responses measured at different luminances and contrasts, extending the results reported by Mante et al. (2005) from the cat LGN to the primate LGN.

### Response gain and saturation for M and P cells at low luminance values

An unexpected finding illustrated in Figure 5 and Extended Data Figure 7-1 is that many M and P cells appear to plateau at a low firing rate for high temporal frequency stimuli under low-luminance conditions. Firing rates saturate at low contrast levels, making response gain measurements more variable. The result is that cells respond less strongly to high temporal frequency stimuli at low luminance compared with high luminance. Prior studies have not reported this interaction between saturation and luminance for high-frequency stimuli. The work of Benardete and Kaplan (1999), which reported a linear contrast response for high-TF stimuli, was conducted using similar retinal illuminances to our highest value. This effect is subtle and, considering the separability measurements discussed above, accounts for only a small fraction of the variance of both P and M cell responses. One caveat to this result is that the decreased responsivity at low luminance levels may make our saturation estimates more vulnerable to noise. It is possible that with additional stimulus presentations, the contrast response function for high-frequency stimuli might appear more nearly linear.

### Alternate sources of response gain variation

The analyses in this article quantify how response gain varies as a function of temporal frequency and luminance in both cell classes. However, two additional variables may account for the variance in response gain we find. The first is the influence of eccentricity in our measurements.

The LGN cells in this study had receptive fields spanning eccentricities ranging from 0.5° to 35° from the fovea, and, as noted in the Introduction, differences in receptive field size between the fovea and periphery might impact response gain. In our sample, there was a weak but significant correlation in P cell response gain with eccentricity (Spearman’s correlation: *r* = 0.33, *p* < 0.005). This effect was subtle and primarily driven by a small population of eight cells recorded >10° from the fovea. Given the limited sample, this trend is inconsequential to the analysis of saturation indices, firing rate, or separability. M cell response gain was uncorrelated with eccentricity (Spearman’s rank correlation: *r* = 0.25, *p* = 0.133).

A second factor that might account for the variation in response gain in either the M or P population is the ON or OFF polarity of the cells we recorded. Prior studies have demonstrated variations in spatial contrast gain control in the LGN and V1 as a function of ON-OFF preference (Kremkow et al., 2014; Archer et al., 2021), and prior reports have reported asymmetries in temporal contrast adaptation for ON and OFF cells (Chander and Chichilnisky, 2001). In our study, we classified each cell as ON or OFF during our initial characterization (see Materials and Methods). However, we did not find a significant difference in the degree of saturation or response gain as a function of contrast polarity. This result is not entirely unexpected based on prior results. Benardete and Kaplan (1999, 1997) did not observe ON-OFF asymmetries in P retinal ganglion cells and for most aspects of M retinal ganglion cells. The exception was a slight difference in the low-pass characteristics of M cells. Chander and Chichilnisky (2001) showed more significant differences in their study of parasol ganglion cell populations. However, they recorded the activity of cells at eccentricities at or beyond 40° of eccentricity, beyond the scope of our measurements, and using different methods (white noise reverse correlation with different rms contrast levels).

### Is separable gain control universal?

Our results in Figures 8 and 9 imply that the independence of luminance and contrast gain mechanisms reported by Mante et al. (2005) in the cat LGN also holds for M and P cells of the monkey LGN. Despite some diversity across and within cell types (Fig. 4), we could decompose temporal frequency tuning into changes driven solely by luminance or contrast. The degree of separability we observed supports the hypothesis that gain control mechanisms reflect the independence of luminance and contrast observed in natural scene statistics (Frazor and Geisler, 2006). Evidence across species suggests that luminance adaptation of the kind we report here begins at the cones and propagates downstream (for review, see Rieke and Rudd, 2009). Contrast adaptation involves changes at multiple sites of the inner plexiform layer of the retina (for review, see Demb, 2008) that are downstream of the cone photoreceptors. The current results show that little interaction between each adaptation mechanism is apparent within the primate LGN. The results imply that the LGN-specific adaptation mechanisms, initially reported in the primate by Kaplan et al. (1987), are uncorrelated with luminance adaptation. This separability of luminance and contrast adaptation is also a property of neurons in the visual cortex of the cat (Geisler et al., 2007).

One methodological limitation to these past studies, including ours, is the range of tested luminances. For example, in the current work, given the limitations of our CRT display, retinal illumination varied between 100 and 1150 Td. While these values are within the photopic range, they fall short of the 300–100,000 Td encountered in natural daylight. Recent results suggest that at these levels, there can be more potent interactions between luminance and contrast, at least in neurons recorded from cat V1 (Rahimi-Nasrabadi et al., 2021).

More sophisticated display systems are needed to surmount these limitations of CRT monitors and measure responses across a broader range of background luminances from the low-scotopic to the high-photopic level. This methodological improvement will be essential to future research on luminance and contrast gain controls from the retina through the cortex.

## Synthesis

Reviewing Editor: Nicholas J. Priebe, University of Texas at Austin Decisions are customarily a result of the Reviewing Editor and the peer reviewers coming together and discussing their recommendations until a consensus is reached. When revisions are invited, a fact-based synthesis statement explaining their decision and outlining what is needed to prepare a revision will be listed below. The following reviewer(s) agreed to reveal their identity: NONE. Note: If this manuscript was transferred from JNeurosci and a decision was made to accept the manuscript without peer review, a brief statement to this effect will instead be what is listed below.

The authors have addressed all the issues previously raised.

One comment arose from the revision, which the authors may wish to address.

The authors may want to clarify how they classified cells into ON and OFF. In the current version of the paper, there is no description of the stimulus used for this classification in the methods section. There is a sentence in the discussion that gives the impression that the ON-OFF classification was not straight forward and required different tests (lines 606-607).
